# GENDER DIFFERENCES IN DEPRESSIVE SYMPTOMS’ IMPACT ON CHRONIC DISEASES, FUNCTIONAL LIMITATIONS, AND MORTALITY

**DOI:** 10.1093/geroni/igae098.3988

**Published:** 2024-12-31

**Authors:** Seoyeon Ahn, Abhijit Visaria, Rahul Malhotra, Angelique Chan

**Affiliations:** Duke-NUS Medical School, Singapore, Singapore; Duke-NUS Medical School, Singapore, Singapore; Duke-NUS Medical School, Singapore, Singapore; Duke-NUS Medical School, Singapore, Singapore

## Abstract

Despite extensive research on chronic diseases, functional limitations, and depressive symptoms at older ages, the patterns of change and interconnectedness among them remain unclear. We examined the trajectories of chronic diseases (cardiometabolic conditions and chronic pain) and functional limitations in individuals aged 60-69 years at baseline and explored how depressive symptoms influenced the onset of chronic diseases, functional limitations, and mortality using data from three waves of the Panel on Health and Aging of Singaporean Elderly (2009-2015; n=386 men and 471 women). We used multichannel sequence analysis to investigate gender-specific health trajectories and random-effects logistic regression to examine depressive symptoms’ role in the onset of chronic diseases, functional limitations, and mortality. Inverse probability of attrition weights were used to account for selection bias due to loss to follow-up. Our findings reveal distinct gender-specific health trajectories. Men had higher mortality rates but experienced slower progression in chronic pain and physical functional decline, despite an earlier onset and higher prevalence of cardiometabolic diseases. Women lived longer but experienced earlier onset and faster progression of chronic pain and functional decline. Depressive symptoms were linked with faster onset of cardiometabolic diseases and functional limitations in both genders, and with mortality only in men. Individuals, families, providers, and policymakers should be mindful of the gender differences in the onset and progression of health deterioration and mortality in old age. Our findings also highlight the need for gender-specific research on the complex interactions between chronic diseases, functional limitations, mortality, and depressive symptoms.

